# Study on the interaction between active components from traditional Chinese medicine and plasma proteins

**DOI:** 10.1186/s13065-018-0417-2

**Published:** 2018-05-04

**Authors:** Qishu Jiao, Rufeng Wang, Yanyan Jiang, Bin Liu

**Affiliations:** School of Chinese Pharmacy, Beijing University of Chinese Medicine, Beijing, 102488 China

**Keywords:** Active components, Traditional Chinese medicine, Plasma protein binding, Research methods

## Abstract

Traditional Chinese medicine (TCM), as a unique form of natural medicine, has been used in Chinese traditional therapeutic systems over two thousand years. Active components in Chinese herbal medicine are the material basis for the prevention and treatment of diseases. Research on drug-protein binding is one of the important contents in the study of early stage clinical pharmacokinetics of drugs. Plasma protein binding study has far-reaching influence on the pharmacokinetics and pharmacodynamics of drugs and helps to understand the basic rule of drug effects. It is important to study the binding characteristics of the active components in Chinese herbal medicine with plasma proteins for the medical science and modernization of TCM. This review summarizes the common analytical methods which are used to study the active herbal components-protein binding and gives the examples to illustrate their application. Rules and influence factors of the binding between different types of active herbal components and plasma proteins are summarized in the end. Finally, a suggestion on choosing the suitable technique for different types of active herbal components is provided, and the prospect of the drug-protein binding used in the area of TCM research is also discussed.

## Introduction

Traditional Chinese medicine (TCM) is the summary of practical experience of Chinese people for thousands of years in the fight against disease. It is the treasure of Chinese culture and constitutes multi-billion-dollar markets—more than 1500 kinds of herbal medicines are sold as dietary supplements or the raw material of medicines [1]. Its active components are the substantial basis for the treatment of various diseases and the related study is also one of the most important parts of the modernization of Chinese herbal medicine.

Generally speaking, the concentration of the free active (or toxic) components is directly related to the biological effect (or poisoning), and the concentration of the free drugs in plasma is directly related to the concentration in the tissue. When drugs are absorbed into the blood, drug-plasma protein binding (PPB) is a common and reversible dynamic process [2]. PPB is one of the important parameters of drug efficacy and safety, and the determination of bound fraction is a necessary step in drug discovery and clinical trials [3]. It determines the pharmacokinetic and pharmacodynamic characteristics of drugs and influences drug absorption, distribution, metabolism, excretion and toxicity (ADMET) [4, 5]. It is generally considered that only free drug can transfer through biological membranes, combine with the appropriate site of action and drive the therapeutic outcome [6]. And then it displays the pharmacological and/or toxicological effects [7]. Small molecular substances can be protected from some elimination pathways, such as enzymatic reactions in the liver or blood and glomerular filtration of the kidneys, by forming non-covalent complexes with plasma proteins [8]. As a drug reservoir, the bound drug fraction can maintain an effective concentration and prolong the duration of the drug action. For the drugs with high affinity for plasma proteins, they generally need a higher dose to reach therapeutic level, have a long half-life and probably increase toxicity. Conversely, the drugs with low plasma protein binding affinities are limited in their ability to perfuse tissues and reach the site of action [9].

Although many Chinese herbal medicines have been proved to be effective by modern clinical trials and pharmacological studies, their active components and the remedial mechanism are still unclear [10]. The pharmacological activities of Chinese herbal medicines are considered to be the combination of multi-components effects, including the interactions of active components with proteins. It is well known that a kind of herbal medicine usually contains hundreds of different components [11]. There is no doubt that this is a complex and heavy work to elucidate the mechanism of action of these components. Therefore, it is extremely valuable to investigate the binding of one or a few active components from Chinese herbal medicine with plasma proteins.

## Plasma proteins involved in drug binding

Major drug-binding proteins in plasma are human serum albumin (HSA), *α*_1_-acid glycoprotein (AAG) and lipoproteins [12]. They have many important physiological functions, for example, mediating osmotic pressure and nutrient delivery, participating in the clot formation and immune response [13]. It is generally accepted that acidic drugs display greater affinity for HSA, while AAG is primarily responsible for the binding of neutral and acidic drugs [14]. HSA, as the most abundant protein in plasma proteins, is in a position to bind endogenous ligands (e.g., fatty acids, amino acids, hormones, bile acids, metals and toxic metabolites) as well as drugs [15–17]. AAG is the second most abundant one, and its endogenous ligands include heparin, serotonin, histamine, steroid hormones and so on [18]. Research reporting on drugs binding to lipoprotein is still sparse.

As the most abundant plasma protein with amazing properties and functions, HSA is the most widely explored protein which is always used as the ligand-biological macromolecules interaction model [19]. Through the first crystallographic analyses of HSA, it is revealed that the protein, as a kind of nonglycosylated molecules, consists of 585 amino acids and 35 cysteine residues, forming 17 disulfides and one free sulfhydryl group at Cys34. The classical researches revealed that the atomic structure of HSA consists of three homologous *α*-helical domains (I–III) each including two subdomains (A and B) [20]. The protein has two high affinity drug binding sites, named as Sudlow’s sites in subdomain IIA and IIIA [8]. Drug site 1 (subdomain IIA) is composed of three extended sub-chambers and a central zone. The inside of the pocket is mainly non-polar molecules. Two clusters of polar residues located in the bottom (*Tyr* 150, *His* 242, *Arg* 257) and the entrance (*Lys* 195, *Lys* 199, *Arg* 218, *Arg* 222) are also identified. Drug site 1 is occupied by phenylbutazone and warfarin. Drug site 2 (subdomain IIIA) is smaller than site 1, but it can accommodate large molecules, such as ibuprofen and thyroxine. In addition, there is another binding site named site 3, to which the digitoxin binds. Because of the structural homology with HSA, bovine serum albumin (BSA) is also a common interaction model used for investigating PPB [21].

## Methods to investigate the interaction between active herbal components and plasma proteins

In recent years, with the development of Chinese herbal medicine, researchers have been paying more and more attention to the pharmacological activity of components in herbal medicine, and numerous experimental techniques have been used in the characterization of PPB. The work has become increasingly diverse and detailed by the application of spectroscopy, chromatography, thermodynamics, electrochemistry and other techniques. The principle and the detection methods of current analysis tool have been introduced in several theses [8, 19, 22]. In this article, a brief introduction of common methods is given, and the applications of techniques used in the investigation of the interaction between active herbal components with plasma proteins are described in detail.

### Membrane technology

#### Equilibrium dialysis (ED)

ED, combined with highly-sensitive assay, such as high performance liquid chromatography (HPLC) and mass spectrometry (MS), is regarded as the gold standard to determine protein binding rate. The working principle of ED is that the small drug molecules could be separated from protein solution by semipermeable membrane. The small drug molecules could pass through the semipermeable membrane until the dialysis reaches equilibrium, while the drug-protein complexes are retained in the dialysis bag. The binding rates of drug molecules with plasma proteins can be calculated by measuring the concentrations of small molecules in the solution on both sides. ED is an easy, economical, practical method and can eliminate the possible effect of non-specific binding [23, 24]. In recent years, ED has been widely used in the multi-component drug research in Chinese herbal medicine. Liu et al. investigated the effects of sinomenine on the therapeutic action of paeoniflorin in the treats-rats by an equilibrium dialysis assay in vitro [25, 26]. The results showed that the protein binding ability is not influenced when they are administrated simultaneously. Wang et al. used a kind of dialysis sampling on-line coupled with HPLC (DS-HPLC) to monitor the interactions of multi-components in danshen (*Salvia miltiorrhiza*) injection with BSA [27]. The five components (danshensu, protocatechuic acid, protocatechuic aldehyde, caffeic acid and ferulic acid) in danshen injection had suitable binding degrees with BSA. Talbi et al. found that wogonin had a very high protein binding degree (over 90%) with rat plasma [28].

But ED also has disadvantages in some ways, including a long time for balancing, the strict control of the pH of plasma and buffer solution, dilution effect and Gibbs–Donnan effects, etc. [19, 29–31]. In recent years, the equilibrium dialysis devices based on the 48-well and 96-well plates have been used in the plasma protein binding studies [32]. The unique design of the device increases the surface area-to-volume ratio and offers the possibility of reducing equilibration times and higher assay throughput. Compared with traditional equilibrium dialysis, this device also has many advantages including easy-to-clean, reusability, the reduction of the drug non-specific absorption and capability of being automated. As the early screening tools for drug research, rapid equilibrium dialysis (RED) device and parallel artificial membrane permeability assay (PAMPA) are two major in vitro models based on Teflon base plate.

The RED device comprises replaceable tube inserts and a 48-well Teflon base plate. Each insert is divided into a buffer compartment (white) and a plasma compartment (red) by a semipermeable membrane at molecular weight cut-off (MWCO) = 8000. Each plate could be sealed with sealing tape and self-adhesive lid. The volume of the insert should be checked to guarantee the little to no volume change occurred [33–35]. Kim et al. developed a RED device combined with LC–MS/MS method to quantify the acacetin in human plasma [36]. The results showed a concentration-independent and extensive protein binding of acacetin in human plasma. The general PAMPA plate system consists of an acceptor compartment (96-well filter plate) and a donor compartment (96-well receiver plate) [37]. Each well of the 96-well microfiltration membrane is filled with 10 μL of the artificial membrane solution which is made of film-forming material dissolved in organic solvent. The 96-well filter plate will be placed on the receiver plate to allow the artificial membrane to touch the donor fluid. And thus the system forms a sandwich structure: the bottom is the donor liquid of the sample, and the drug molecules diffuse from the donor tube into the upper receptor tube through the artificial membrane. When the diffusion is completed, the receptor fluid and the donor fluid can be used to make quantitative analysis [38, 39]. Singh et al. investigated the blood uptake characteristics, protein binding, pharmacokinetics and metabolism of formononetin by this system. Formononetin had high protein binding rate, and the rapid absorption of which might due to the high permeability and lipophilicity [40].

#### Ultrafiltration

Ultrafiltration is a popular alternative of ED and a better choice for the clinical pharmacokinetic and pharmacodynamic studies of new drugs [41]. Similar to ED, it utilizes semipermeable membrane to separate the device into two chambers. Driven by the pressure difference or centrifugation (approximately 2000×*g*), the drug molecules diffuse through the semipermeable membrane. Because this method achieves the rapid separation of small molecules in plasma, the work efficiency is greatly increased [42]. Ultrafiltration is more suitable for highly lipophilic compounds, and it, in combination with HPLC, GC–MS, LC-IT-TOF–MS, RRLC-ESI–MS–MS and other high sensitivity detection methods, has been applied to determine the plasma protein binding rate of active herbal components [43–50].

In ultrafiltration, the concentration polarization, which is caused by the diffuse direction of the small molecules, is perpendicular to the ultrafiltration membrane. It will compromise the protein-binding equilibrium and affect the determination of free drug concentration. Li et al. developed a novel and practical method based on hollow fiber centrifugal ultrafiltration (HFCF-UF) combined with HPLC to determine the plasma protein binding of three coumarins in human plasma [51]. The device was made of a glass tube, in which a U-shaped hollow fiber was placed. Therefore, the direction of molecular diffusion was completely parallel to the membrane. The binding rates of bergenin, daphnetin, and scopoletin determined by this method were 52.7–53.5, 56.7–58.0 and 59.0–60.1% respectively, which were consistent with the results of the equilibrium dialysis method. Compared with the classical method, HFCF-UF has higher precision and accuracy and simpler sample preparation procedure.

#### Microdialysis

Microdialysis was originally used to determine the free adenosine levels in the brain of rats [52]. In recent years, it has become an important technique for direct determination of the free drug concentration in the body’s plasma, tissue and other physiological fluids. The key of this technique is the probe with a semipermeable membrane which has a molecular mass cut-off ranging from 5000 to 50,000 Da [53]. The biggest advantage of microdialysis is the real-time sampling and on-line analysis in a condition that hardly interfered with the normal life activity of animals [54]. With this method, we can continuously measure the concentration of unbound drug over time in vivo [55]. Another advantage of microdialysis is the convenience for automation that hyphenated with many sensitive analytical techniques like HPLC, capillary electrophoresis (CE), nuclear magnetic resonance (NMR), etc. [56].

Microdialysis has many features in the field of traditional Chinese medicine. The most prominent feature is the ability to simultaneously investigate the interaction of multi-components in Chinese herbal medicine or compound prescription with plasma proteins, and thus finding the potential active components [57]. Qian et al. found that chlorogenic acid, luteolin-3-*O*-glucoside and 4,5-di-*O*-caffeoyl quinic acid might compete for the same binding sites and caffeic acid and rutin had synergistic effects in Flos Lonicerae Japonicae [58]. Wen et al. found that four compounds (chlorogenic acid, calycosin-7-*O*-β-d-glucoside, ferulic acid and calycosin) in Danggui Buxue Decoction had suitable binding degrees with human plasma proteins [10]. These compounds had been proven to be the active components in the prescription. Guo et al. found that compound I and compound M identified in Rhizoma Chuanxiong had the similar binding degrees to HSA as two known active compounds, ferulic acid and 3-butylphthalide [59]. They thought compound I and compound M might be the potential active compounds. The online coupling of microdialysis with sensitive and selective analytical systems has great value and potential in screening the effective components from Chinese herbal medicine.

### Centrifugation

Other than the membrane techniques like ED and UF, ultracentrifugation (UC) techniques separate the free drug molecule from the drug-protein complex by high gravitational force (625,500 g). Small molecules and proteins have different density or sedimentation rate in centrifugal force field. After centrifugation, the drug molecules combined with high density plasma macromolecules will rapidly subside to the bottom, while the free fraction can be quantitated in the supernatant of the centrifuge tube [8, 60]. UC has several advantages such as the lack of Gibbs–Donnan effects and nonspecific adsorption, adoptability for high molecular weight and lipophilic compounds [61]. But the limit factors, like the expensive equipment and the low throughput caused by the relatively smaller number of samples that can be processed at one time, restrict the application of UC techniques. Li et al. removed the plasma proteins by ultracentrifugation and measured the concentration of syringopicroside in serum by HPLC after injection of low, medium and high doses [62]. The results showed that syringopicroside was a medium plasma protein binding drug and the binding rate was not dependent on the doses.

### Extraction methods

#### Solid phase microextraction (SPME)

SPME is a simple and effortless technique to determine free drug concentration [63]. It was developed as a convenient method for volatile organic compounds in the early 1990s. Because of its simplicity, SPME has been used to monitor the metabolites, ligand–protein binding, toxicity and permeability of drugs, and metabonomics of volatile or semivolatile compounds. Basic theory of this technique is that the solid support, which is hydrophobic and dispersed with extracting phases, is exposed to the test sample for a definite period of time [64]. Then, the enriched drug molecules in the extraction phase are rapidly and completely separated into the analytical instruments by high temperature or solvent elution methods. SPME fiber is an optical glass fiber which is evenly coated with a polymer coating [65]. Because of the non-depleting extraction mode, SPME is a particular suitable technique for drug-protein binding studies [66]. The development of biocompatible coating makes SPME can investigate complex biological samples for any binding equilibriums [64, 67]. The relative high accuracy and sensitivity, no need to use organic solvents and possibility to automate are the main advantages of SPME. But the fouling formed of protein-fiber binding may lead to erroneous estimate of the concentration in the fiber coating [63, 65].

SPME has been used in investigating the interaction between active components in TCMs and plasma proteins [68–70]. Volatile oil widely exists in traditional Chinese medicine derived from plants. It is well known that there are 136 genera of 56 families in China containing volatile oil. In addition to volatile oil, there are many aromatic substances of Chinese medicine, such as musk, bezoar and borneol. These components are complex, volatile and insoluble in water. Therefore, conventional methods are difficult to determine the binding degrees of these components with plasma proteins. Headspace-SPME, in which the extraction fiber is placed in the upper space of the samples, is more suitable for the determination of these components. The extraction head of headspace-SPME does not touch the sample, and thus avoids the matrix effect. Hu et al. developed a headspace negligible-depletion extraction mode (nd-SPME) coupled to GC method to investigate the noncovalent interaction of borneol with HSA [71]. The method was simple, sensitive, rapid and could overcome the drawback of losing volatile components in the binding or transfer process.

#### Hollow fiber liquid–liquid phase microextraction (HF-LLPME)

HF-LLPME is an inexpensive sample preparation method to investigate the drug-protein binding under physiological conditions without disturbing the equilibrium between drugs and proteins [72]. In microextration system, the polypropylene hollow-fiber membrane is filled with 15–25 μL of extraction solvent and placed into the mixture of drug and protein. When small molecule drugs establish distribution equilibrium between bulk aqueous phase and organic phase, the unbound concentration of drugs can be determined by analytical instrument [73, 74]. This method allows simultaneous determination of multi-components. Compared with the traditional liquid–liquid extraction (LLE), HF-LLPME allows the sample under vigorous stirring conditions and requires less organic solvents. Therefore, the method reduces the analysis time of drugs transferred across the membrane. HF-LLPME has potential to determine drug-protein binding of active components from TCMs in the complex sample matrices. Hu et al. investigated the interaction of four furocoumarin and two alkaloid compounds with BSA by HF-LLPME combined with HPLC [75, 76]. The results demonstrated that HF-LLPME is a simple, rapid and effective method for characterizing drug-protein binding parameters without separation.

### Chromatographic methods

#### High performance affinity chromatography (HPAC)

HPAC is a kind of adsorption chromatography which uses a biologically related agent as stationary phase [77]. As one of the most effective methods that separate and purify the biological macromolecules, HPAC is based on the specific reversible interaction between the target protein and the immobilized ligand. HPAC immobilizes the proteins onto a support and injects the interacting solute into the column. The drugs with high affinities will be eluted later than low-affinity drugs because of the strong interaction [78]. The method has been coupled with HPLC to determine the binding of drugs and various proteins such as HSA, AGP and lipoproteins in plasma [79]. Many reports have demonstrated that the allosteric interactions and displacement effects seen on HSA columns are similar to those observed for soluble HSA [80, 81]. Compared to the traditional methods, HPAC has many advantages such as automation, high precision, speed, specificity and the ability to work with small amounts of a target solute [82, 83]. But some problems still need to be solved, such as the short service life of the column and the high standards of the preparation of fillers.

For complex research objects, such as Chinese herbal medicines, HPAC could eliminate the interference of a large number of inactive impurities due to the specificity and selectivity of the stationary phase in combination with the active component. Cai et al. detected the binding rates of puerarin and goitrin with HSA by a HSA column [84]. The results were consistent with those obtained by ultrafiltration method and demonstrated that HPAC method was a reliable technique. HPAC is often applied to investigate the competition displacement in different active herbal components with plasma proteins. Lei et al. investigated the competition interaction of ferulic acid and paeonol with HSA by HPAC [85]. The results demonstrated that ferulic acid and paeonol competed for binding to the indole site (site 2) and the main force was deduced to be hydrogen bonding according to the thermodynamic parameters.

#### Capillary electrophoresis (CE)

CE is a series of related techniques that the separation processes are happened in narrow bore capillaries under the force of electric field [86]. It is a powerful analytical tool that is widely used in the analysis of small organic molecules, inorganic ions and biopolymers [87]. In the years past, CE has become a hit for drug-protein interaction measurements because of low sample requirements and consumption, simplicity, short analysis times, high sample throughput and high separation efficiencies [5, 88]. There are several modes of electrophoresis to investigate the drug-protein binding, including affinity CE (ACE), vacancy peak (VP), Hummel–Dreyer method (HD), frontal analysis (FA) and zone migration CE (CZE) [89]. Among them, ACE, FA and CZE have the same advantages: (1) only a small number of proteins and drugs are required; (2) all interacting components can be investigated in free buffer solution at physiological conditions; (3) binding constants of multi-components can be simultaneously estimated. Therefore, these methods are suitable for the study of some Chinese herbal medicines which are chemically complex and expensive [90–93].

In recent years, with the development of microdialysis in the field of medicine, CE combined with microdialysis techniques has been used in pharmacokinetics research [94, 95]. It combines the characteristics of continuous, dynamic sampling in microdialysis and less sample volume in CE. The method could objectively analyze the drug-protein binding behavior of specific drugs under physiological and/or pathological conditions. Although there are few reports about the research on CE combined with microdialysis techniques in the field of TCMs, there is no doubt that it is the best choice if you want to study the change of multi-components in Chinese herbal medicine and plasma protein binding in disease states.

#### Sodium dodecyl sulfate polyacrylamide gel electrophoresis (SDS-PAGE)

SDS-PAGE, which was proposed by Laemmli in 1970, is a charming and powerful tool for protein characterization [96]. The principle of SDS-PAGE is the positive correlation between electrophoretic mobility of protein and the molecular mass [97–99]. SDS, a kind of anionic detergent, could denature original proteins, eliminate protein’s original surface charge and destroy the structure. And then the SDS-protein complexes are formed. The advantages of SDS-PAGE are simplicity, less analysis time and excellent repeatability. However, because of the large errors and low resolution of SDS-PAGE, the method cannot reflect the binding degree of drug and the application is rare. Kaldas et al. identified the irreversible binding between oxidized quercetin and protein by a radioactively labelled drug and SDS-PAGE. The result showed that quercetin oxidized by hydrogen/peroxidase covalently links to proteins and with particularly high affinity for HSA [100].

### Spectroscopic methods

#### The main spectroscopic methods of the interaction between active herbal components and plasma protein

Spectroscopic methods are based on the change of spectroscopic properties of proteins in ligand–protein binding processes. The information of drug-protein binding can be obtained without separation.

##### Fluorescence spectroscopy

Fluorescence spectroscopy is the most widely used and powerful spectroscopic technique for gaining the information about the binding of drug and plasma proteins because of its accuracy, sensitivity, rapidity and usability [101, 102]. Because of the existence of aromatic, such as tryptophan (*Trp*), tyrosine (*Tyr*) and phenylalanine (*Phe*), serum proteins are considered as endogenous fluorescent substance. When 295 nm is selected as the excitation light source, endogenous fluorescence is all from the *Trp* residue [103]. When small molecule drugs interact with proteins, they are often able to decrease the fluorescence intensity or quench the intrinsic fluorescence of proteins. Synchronous fluorescence spectrum, which can be obtained by simultaneous scanning excitation wave and emission wave, could determine the emission spectra of *Ty*r and *Trp*. Three-dimensional fluorescence spectroscopy, a kind of new fluorescence analytical technologies developed in the past 20 years, can visually show the microenvironment and conformational changes under different conditions of *Trp* in protein molecules.

##### UV–Vis absorption spectroscopy

UV–Vis absorption spectroscopy is another widely used technique to investigate drug-protein binding. Inherent ultraviolet absorption of plasma protein is mainly due to the absorption of light generated by the n–π* transition in indolyl group of *Trp*, the phenol group of *Tyr* and the phenyl group of *Phe*. The changes of the peak intensity and position of two characteristic absorptions could reflect the conformational change of proteins.

##### Fourier transform infrared spectroscopy

Fourier transform infrared spectroscopy (FT-IR) is one of the popular techniques for the structural characterization of proteins. The most important advantage of FT-IR compared with other methods is the extensive applicability of any biological system in a wide variety of environments [104, 105]. The characteristic absorption peaks of amide groups of proteins are the most valuable ones for the study of protein secondary structure.

##### Circular dichroism spectroscopy

Circular dichroism (CD) spectroscopy is based on the different absorption of the left and the right circularly polarized light by optically active groups of proteins. The CD spectra of serum proteins are generally divided into two wavelength ranges—178–250 nm for the far-ultraviolet CD spectrum and 250–320 nm for the near-ultraviolet CD spectrum. Extrinsic Cotton effect is used to represent the change of the normal CD spectrum in the binding of ligands to HSA. The far-ultraviolet CD spectrum, the most commonly used spectrum in protein study, could reflect the protein secondary structure information. The peak in the near-ultraviolet region is sensitive to reflect the subtle changes in the conformation of the serum protein.

##### Surface plasmon resonance (SPR)

Surface plasmon resonance (SPR), which can monitor the formation and dissociation of the drug-protein complex in real-time and obtain the equilibrium (*K*_D_) and kinetic (*k*_on_ and *k*_off_) data for the interaction, is one of the most excellent optical biosensor technologies [106–108]. The conventional SPR device requires a biomolecule to be immobilized on a sensor chip. The sensor chip can monitor the change of refractive index that occurs at the surface of the complexes during form or break process in the binding reaction [108–110]. Another partner in solution is placed together with the sensor. Fabini et al. developed a sensor chip whose serum albumins were covalently bound to the carboxymethyl dextran layer of the sensor chips through its primary amine groups by an amine coupling reaction [111]. The result indicated that cucurbitacins were able to modulate the binding of biliverdin and serum albumins.

Compared with the traditional analytical methods or means, SPR has much salient features such as free label detection, real-time dynamic analysis, non-destructive testing, high sensitivity and larger detection range [112, 113]. Shi et al. developed a rapid, continuous and effective method to identify the multi components from Radix Astragali which were bound to HSA by a SPR-HPLC–MS/MS system [114]. The data of reverse ultrafiltration assay showed a good agreement with SPR. SPR has become a popular technique to study DNA–DNA, antibody-antigen, protein–protein interaction and the interaction between drugs and specific cellular receptor proteins, key genes, proteases and other disease-related biomolecules.

Besides, there are several commonly used spectra like mass spectrometry (MS), nuclear magnetic resonance (NMR) spectrum, resonance light scattering (RLS) and surface-enhanced Raman scattering spectroscopy. Several spectra are generally used together to study the drug-protein binding and could give more comprehensive data and results.

#### The main research contents of spectroscopic methods of the interaction between active herbal components and plasma protein

What we can learn from the result of the spectroscopic methods about the binding between plasma proteins and active herbal components include judging the mechanisms of fluorescence quenching, calculating the binding constant, the number of binding sites, the distance between *Trp* and drug molecule and thermodynamic parameter, determining the binding site, binding forces and change of protein’s secondary structure, etc.

##### Mechanisms of fluorescence quenching

The effect of active herbal components on the intrinsic fluorescence of serum albumin can be divided into fluorescence quenching and fluorescence sensitizing. In most cases, fluorescence quenching is the main one. The mechanism of fluorescence quenching can be classified as dynamic quenching and static quenching. The reason for the static quenching is the formation of non-fluorescent complex between the fluorescent molecules in the ground state and quencher [101]. So that the fluorescence spectra of the static quenched fluorescent molecules change. The dynamic quenching is caused by the collision of the fluorescent molecules in the excited state with the quencher. After collision, the fluorescent molecules return to the ground state, so that the fluorescence spectra of the dynamic quenched fluorescent molecules do not change.

The mechanism of fluorescence quenching can be determined by the following points [115].

Firstly, in the Stern–Volmer equation, the value of *K*_*q*_ is about 10^9^–10^10^ L (mol s)^−1^. If *K*_*q*_ calculated from *K*_*sv*_ and *τ*_0_ is much larger than this range, it means that the binding is not diffusion control and the mechanism of fluorescence quenching is static quenching. Conversely, the mechanism may be dynamic quenching. The *K*_*q*_ of delphinidin-3-*O*-glucoside at 298 K was 6.163 × 10^12^ L mol^−1^s^−1^, which was much higher than the maximum diffusion collision quenching constant value (2.0 × 10^10^ L mol^−1^s^−1^). It illustrated that the interaction of delphinidin-3-*O*-glucoside with BSA occurred by the static quenching [116].

Secondly, when the dynamic quenching occurs, the UV–Vis absorption spectra of fluorescent molecules do not change. In the event of static quenching, the changes occur on the UV–Vis absorption spectra of fluorescent molecules. HSA had an absorption peak approximately at 280 nm on the UV–Vis absorption spectra. The increasing neohesperidin dihydrochalcone concentration decreased the absorption peak of HSA and a slight blue shift could be observed. These evidences showed that the interaction between neohesperidin dihydrochalcone and HSA belonged to static quenching [117].

Thirdly, dynamic quenching relies on molecular diffusion. The temperature rise increases the diffusivity of the molecules and the possibility of molecular collision. So the quenching constant increased with temperature. On the contrary, the increase of temperature may reduce the stability of non-fluorescent complex, thereby reducing the degree of static quenching. The value of *K*_*sv*_ of ferulic acid was 3.818 × 10^4^, 3.912 × 10^4^, and 4.881 × 10^4^ at 25, 35 and 45 °C. The trend that the quenching constant increased with the increase of temperature indicated that the interaction of ferulic acid with HSA was influenced by diffusion [118].

And, fourthly, in the case of static quenching, quenching does not change the lifetime of the excited state of fluorescent molecules: τ_0_/τ = 1. Whereas in the case of dynamic quenching, the presence of the quencher reduces the lifetime of fluorescence: τ_0_/τ = *F*_0_/*F*. Yang et al. found that the increasing concentration of paclitaxel hardly changed the lifetime of HSA (from 5.58 to 5.47 ns) and the quenching followed a static mechanism [119].

But for some active herbal components, the static and dynamic procedure may exist simultaneously. Cheng et al. investigated the interaction of tetrandrine with BSA and HSA. The trend that the values of *K*_*sv*_ increased with the increasing temperature indicated that the interaction belonged to dynamic quenching [120]. But the UV–Vis spectra data and the higher *K*_*q*_ ~ 10^13^  L mol^−1^s^−1^ at 298 K showed the formation of complex. Therefore, a combination of the static and dynamic quenching played an important role in the interaction of tetrandrine with BSA and HSA. Similarly, Gao et al. found an increase of absorbance band intensity on the UV–Vis spectra when the concentration of syringin was increased in HSA [121]. However, the value of *K* increased with the increasing temperature. Therefore, they thought that the quenching mechanism of HSA by syringin was dynamic quenching, while static quenching could not be ignored.

##### Binding constant and the number of binding sites

Binding constant and the number of binding sites can be calculated by Stern–Volmer equation, modified Stern–Volmer equation, Lineweaver–Burk equation, Benesi–Hidebrand equation, Benesi–Hidebrand equation and multiple binding sites equation. Stern–Volmer equation is the most well-known formula which is used to calculate binding constant and the number of binding sites and could apply to study the fluorescence quenching mechanism. Both static quenching and dynamic quenching process follow this equation [19]. Modified Stern–Volmer equation could reduce the effect of other light in the fluorescence experiment on the measured value [122, 123]. When the linearity of the Stern–Volmer equation is not ideal, the Lineweaver–Burk equation can be used. But Matei et al. predicted slightly higher *K* values by this model than classical Scatchard equation in the investigation of the kaempferol-HSA complex [124]. They thought that in fact here *K* represented quenching constant which was used to describe the binding efficiency of the quencher to the fluorescent molecules, but not the binding constant. This equation applies to the system with only one binding site. If the small molecule ligand has fluorescence, its fluorescence intensity increases as it interacts with the protein. Bhattacharya et al. modified the Benesi–Hidebrand equation to escape this interference [125]. This equation is suitable for the active herbal components which have auto-fluorescence [126]. For the multiple binding sites system, Zhang et al. proposed a multiple binding sites equation that could calculate the binding constant and the number of binding sites at the same time [127]. The binding constants and the number of binding sites of N-trans-p-coumaroyltyramine, 3-trans-feruloyl maslinic acid, four flavonoid aglycones (baicalein, quercetin, daidzein, and genistein) and their monoglycosides (baicalein, quercitrin, daidzin, and puerarin, genistin) were all calculated by this equation [128–130]. It is noteworthy that all the active herbal components using this equation must follow static quenching.

##### Thermodynamic parameter and binding forces

The binding forces between small molecules and proteins include hydrophobic interactions, electrostatic interactions, hydrogen bonds and van der Waals forces [131]. According to the thermodynamic parameters, the type of binding forces can be roughly determined. The change in enthalpy (*∆H*) can be considered as a constant when the temperature changes a little. Then the values of enthalpy changes and entropy changes (*∆S*) can be calculated from van’t Hoff equation. Ross et al. thought that the type of binding forces can be determined by the sign and magnitude of the thermodynamic parameter [132]. The relationship between thermodynamic parameters and binding forces are shown in Table 1.

**Table 1:** The relationship between thermodynamic parameters and binding forces

Thermodynamic parameter	Binding force
∆*S *> 0	May be hydrophobic and electrostatic interactions
∆*S *< 0	May be hydrogen bonds and van der Waals forces
∆*H *> 0, ∆*S *> 0	Hydrophobic interactions
∆*H *< 0, ∆*S *< 0	Hydrogen bonds and van der Waals forces
∆*H *≈ 0 or very small, ∆*S *> 0	Electrostatic interactions

However, the structure of HSA is very complex and usually there are multiple forces between small molecules and proteins in the actual reaction system. For example, corresponding thermodynamic parameters about the interaction between HSA and icariin were calculated according to van’t Hoff equation [133]. The negative ∆*H* and ∆*S* were the evidence of van der Waal’s force and hydrogen bonds in low dielectric medium. The negative ∆*H* was associated with electrostatic interactions. Therefore, electrostatic interactions cannot be excluded from the binding forces.

##### The distance between Trp in protein and drug molecule

Fluorescence resonance energy transfer (FRET) is the distance-dependent interaction that occurs between molecules with different electronic excited states. According to the Förster’s non-radiative energy transfer theory, two molecules must meet the following conditions: (1) the energy donor can produce fluorescence; (2) UV–Vis absorption spectra of the energy acceptor and fluorescence emission spectra of the energy donor increasingly overlap; (3) the distance between donor and acceptor is less than 7 nm [134]. Because the endogenous fluorescence of protein is mainly produced by *Trp* residue, the distance between the binding site of the drug and the *Trp* residue can be calculated by the Förster’s non-radiative energy transfer theory. This theory is widely used in the study of active herbal components-HSA interactions [135–137].

##### The change of protein’s secondary structure

The binding process of small molecules and proteins may affect the conformation of proteins. The main techniques to determine the effect of small molecules on the secondary structure of proteins contain UV–Vis absorption spectroscopy, synchronous fluorescence spectroscopy, CD spectroscopy and Fourier transform infrared spectroscopy.

###### UV–Vis absorption spectroscopy

When the structure or environment of protein changes, the environment and conformation of the chromophore will also change. And these changes can be expressed through the absorption spectra. By comparing the changes of UV–Vis absorption spectra before and after the binding of the active herbal components and HSA, it is possible to determine the presence of the chromophore in the vicinity of the binding site and the change of microenvironment around protein. For example, apigenin has a strong absorption peak at 202 nm on the UV–Vis spectra [138]. With the increasing of HSA, the position of peaks shifted from 202 to 224 nm and the absorption intensity decreased. It suggested that quercetin interacted with HSA in ionic form in non-planar conformation, and the binding changed the microenvironment around quercetin.

###### Synchronous fluorescence spectroscopy

Synchronous fluorescence spectra can simultaneously scan the excitation and emission wavelengths. The spectral characteristics of a certain amino acid residue can be shown by selecting the appropriate wavelength interval (∆*λ*). The synchronous fluorescence spectra of ∆*λ *= 15 nm and ∆*λ *= 60 nm represent the characteristics of *Tyr* residues or *Trp* residues of HSA. The maximum absorption wavelengths of residues are related to the polarity of their environment. Therefore, the change of the conformation of the protein can be judged by the absorption wavelength [138]. Cheng et al. found that significant red shift of the maxima emission wavelength of *Trp* and *Tyr* residues when adding tetrandrine to HSA and BSA solution [120]. It indicated that the polarity around the *Trp* and *Tyr* residues increased and the hydrophobicity decreased. However, the microenvironment changes of *Trp* and *Tyr* residues are not necessarily synchronized. Hedge et al. investigated the molecular environment in the vicinity of a chromophore in the presence of hesperitin [123]. A marginal red shift (from 288 to 290 nm) could be observed at ∆*λ *= 60 nm, while the emission maximum did not exhibit a significant shift at ∆*λ *= 15 nm. It indicated that the microenvironment around *Tyr* residue was not affected. But the polarity around the *Trp* residues increased and the hydrophobicity decreased.

###### Fourier transform infrared spectroscopy

The amide bands of protein secondary structure showed a characteristic absorption peak on FT-IR. Among the amide bands of the protein, amide I band is ranged from 1600 to 1700 cm^−1^ (mainly C=O stretch) and amide II band is at 1550 cm^−1^ (C–N stretch coupled with N–H bending mode) [139]. The amide I band is more sensitive to the change of protein secondary structure and more commonly used to test the change of the HSA secondary structure [140]. The assignments of spectral peaks are attributed as follows: 1610–1640 cm^−1^ to *β*-sheet, 1640–1650 cm^−1^ to random coil, 1650–1658 cm^−1^ to *α*-helix, and 1660–1695 cm^−1^ to *β*-turn structure [109]. The absorption peaks of the infrared spectrum often overlap each other to form a broad peak. And the broad infrared bands in the spectra of protein can be analyzed in detail by using second-derivative and deconvolution procedures. The percentage of each secondary structure of protein can be calculated based on the integrated areas of the component bands in amide I [141]. Tang et al. investigated the binding of glycyrrhetinic acid and HSA by multispectroscopic techniques [142]. The FT-IR spectra showed that the peak positions of amide I bands shifted from 1656.40 to 1637.83 cm^−1^ in HSA infrared spectrum after interaction with glycyrrhetinic acid. It demonstrated that the secondary structures of the HSA had been changed after the binding of glycyrrhetinic acid and HSA. The *α*-helix structure reduced from 50.93 to 24.73%, *β*-turn increased from 23.61 to 25.27% and random coil appeared (13.98%).

###### CD spectroscopy

The CD spectra of protein have two negative bands at 208 and 222 nm, which is the characteristic feature of *α*-helix structure. The results of CD spectra could be expressed as MRE (mean residue ellipticity) in deg cm^2^ dmol^−1^ and the percentage of *α*-helix can be calculated by equation [143]. By measuring the percentage of *α*-helix, the conformational change of protein could be determined clearly. Liu et al. investigated the impacts of baicalin and rutin on the interaction between curcumin and HSA [144]. The CD spectra showed that curcumin induced a slight decrease in the *α*-helical content of HSA in the absence and presence of rutin and baicalin, corresponding to a reduction of 3.27, 8.94 and 4.81%, respectively. It demonstrated that the effects of curcumin on HSA were slightly less than those of rutin and baicalin.

##### Binding site

Some fluorescent probes have specific binding to different regions of the HSA, and the binding sites can be determined by the displacement binding experiments using some probes. Commonly used florescent probes include: warfarin, phenylbutazone for site 1; ibuprofen, naproxen for site 2 and digitoxin for site 3. Miklós Poór et al. compared the affinity to HSA between flavonoids and warfarin [145]. They found that different flavone (acacetin, chrysin, apigenin, luteolin), flavonol (galangin, quercetin), and flavanone (naringenin, hesperetin) could displace warfarin and highlighted that flavonoids were powerful competitors for HSA and could bind to the drug site 1. In the competition experiments with ibuprofen probes and warfarin probes for HSA binding sites, Bari et al. demonstrated that quercetin primarily binds to the site located in the subdomain IIA [146].

Displacement binding experiments also can be a guidance to reasonably predict clinical toxic and side effect of active herbal components. Soligard et al. used purified Chinese herbal constituents and sulfisoxazole to displace the bilirubin from HSA from jaundiced newborns [147]. The positive inhibitor control sulfisoxazole increased plasma unbound fraction by an average of 60%, while, no displacement phenomena that neferine, sinomenine, tetrahydropalmitine and notoginsenoside showed up. This experiment revealed that four purified Chinese herbal components possessed no significant potential to increase the sulfiisoxazole concentration in jaundiced newborn infants.

### Electrochemical methods

Electrochemical method, which has characteristic of quick response, easy operation and relatively high sensitivity, provides an important tool for the study of protein bioelectrochemistry [148, 149]. As a commonly used electrochemical method, cyclic voltammetry (CV) detects the current signals of the electrochemical substance which is consumed and/or generated during the biological and chemical interaction of the bioactive material and the substrate [150–153]. The method can use mercury, gold, platinum, glassy carbon, carbon fiber microelectrodes, chemically modified electrodes and so on. Based on the analysis of the changes of position, current and number of redox peak, the stoichiometry of the interaction process and the stability constant of supramolecular compounds can be measured, and the binding mode of small drug molecules and proteins can be assumed.

Electrochemical methods can be used to study the molecules whose absorption spectra are weak, or overlaps occur between their electron transition band and the absorption spectrum of the macromolecules themselves. Cyclic voltammetry provides a possibility for the measurement of these molecules, but it is limited to a certain degree by electrical activity [154]. Ni et al. investigated the interaction of quercetin with BSA by UV–Vis absorption spectrometry, fluorescent spectrometry and cyclic voltammetry [155]. The oxidation peak moved from 465 to 520 mV and the reduction peak moved from 430 to 400 mV. Corresponding data calculated by equation showed that a 1:1 quercetin-BSA fluorescent complex was formed, but this complex did not appear to be electroactive. That could be due to the electroactive parts of quercetin, the 3′- and 4′-OH group, were embedded within the BSA, and this prevented its interaction at the electrode surface and therefore its participation in the redox reaction.

### Calorimetry

Calorimetry is the primary source of thermodynamic information which is produced from the heat exchange of any physical, chemical and biological processes. Therefore, calorimetry has become one of the effective tools for studying in many fields of technology and science [156]. Calorimetry could get the basic physical forces that characterize the binding of drug molecule and protein in detail by measuring heat quantities or heat effects. This method can be used as the verification of the results of spectroscopy, which more accurately reflect the binding of active herbal components and plasma proteins. The application of microcalorimetry, including isothermal titration calorimetry (ITC) and differential scanning calorimetry (DSC), makes the calorimetry develop in the direction of high sensitivity and high accuracy.

ITC is the straightest path to complete the thermodynamic characterization of protein interaction without the requirement for chemical modification or labeling [157]. This advantage sets the technique apart from fluorescence spectroscopy, because fluorescence methods often need a quencher to label proteins. Typically, a syringe containing the ligand is titrated into the cells containing the protein solution. With the formation of ligand–protein complex, binding affinity can be evaluated by monitoring the heat that quantitatively occurs in the release and absorption of the binding process [158, 159]. These experimental data can be fitted into an equation, and the binding constants (*K*_b_), reaction stoichiometry (*n*) and thermodynamic parameters, including molar calorimetric enthalpy (Δ*H*_obs_), heat capacity (Δ*C*_p,obs_), entropy (Δ*S*_obs_) of binding and change in free energy (Δ*G*), can be determined accurately [156, 157]. Zhao et al. developed an ITC combined with CD and UV–Vis spectra method to investigate the interaction of colchicine with HSA [160]. The standard enthalpy of the first class binding site was 29.35 ± 0.36 kJ mol^−1^ (endothermic process). It indicated that the binding of drug molecules and ligand molecules destroyed the hydration layers. Δ*H*_0_ of the second binding site of HSA was − 19.62 ± 0.28 kJ mol^−1^. It showed the main driving force of the binding was hydrophobic interaction. The thermodynamic parameters showed that the first-class of binding process was primarily driven by entropy and the second-class of binding was driven by enthalpy and entropy. Li et al. presented a new and efficient method of using ITC combined with fluorescence spectroscopy, UV–Vis absorption spectroscopy and Fourier transform infrared (FT-IR) spectroscopy, to study the interaction between (+)-catechin and bovine serum albumin (BSA) [161]. Corresponding thermodynamic parameters suggested the binding was synergistically driven by enthalpy and entropy.

But ITC cannot study very high and low affinity process and the experiments that need a great deal of accurate measurements. Differential scanning calorimetry (DSC) is a complementary technique which could be used to investigate the interaction that is not amenable to analysis by ITC. DSC is developed to investigate thermally induced transitions, especially the conformational transitions of proteins [163]. It can measure the heat difference between a sample and a reference substance at the programmed temperature [163]. The changes of protein structure can be estimated from the relevant thermodynamic parameters, such as phase transition temperature, enthalpy and half-width of the lipoprotein, which could get from DSC curve [164]. Khan et al. investigated the effect of berberine and palmatine on the thermal stability of BSA and HSA by DSC [165]. The melting temperature of BSA and HSA by berberine and palmatine were decreased by (6.00 and 6.02) and (5.70 and 6.01) K, respectively, under saturating conditions. It indicated that the binding destabilized the protein structure. Similarly, the effects of sanguinarine iminium and alkanolamine on the thermal stability of HSA were investigated by DSC, too [166].

## Research achievements on the interaction between active herbal components and plasma proteins

In recent years, the study of the interaction between active herbal components and plasma proteins has been a hot spot. The researchers have carried out a lot of investigations and already gained some achievements in this field. According to the type of compounds, the active herbal components are divided into flavonoids, alkaloids, triterpenes, phenylpropanolds and other phenolic substances. Corresponding binding parameters between different types of active herbal components and plasma proteins are shown in Table 2.

**Table 2:** Binding parameters for the interaction between active components in TCMs and plasma proteins

Type	Compounds	Protein	*K* (M^−1^)	Stoichiometry (*n*)	Quenching mechanism	Binding site	*r* (nm)	Driving force	References
	Flavone	BSA	2.139 × 10^4^	2.26		IIA		a, b	[91]
	Rutin	HSA	3.8 × 10^4^	1.43	Static	IIA		a, b	[167]
	Quercitrin	BSA	4.13 × 10^3^	2.32		IIA		b, c	[91]
	Kaempferol	HSA	2.89 × 10^5^	0.65	Static	IIA	2.7	c	[124]
	(+)-catechin	BSA	1.565 × 10^4^	2.17	Static	IIA		a, b	[162]
	Loureirin A	HSA	1.291 × 10^5^	0.88	Static	IIA	2.66	c, d	[168]
	Cochinchinenin C	HSA	2.532 × 10^5^	0.78	Static	IIA	2.975	c, d	[168]
	Hesperitin	HSA	1.29 × 10^5^	1.03	Static	IIA	1.978	a, c	[123]
	Diosmetin	HSA	1.18 × 10^5^	0.84	Static	IIA	3.54	a, c	[136]
	Apigenin	BSA	6.978 × 10^6^	1.266	Static	IIA	1.89	a, c	[169]
		HSA	9.83 × 10^4^	0.8774	Static	IIA	3.21	a, b	[138]
	Farrerol	HSA	4.76 × 10^5^	0.46	Static	IIA	2.63	a, c	[170]
	Baicalein	HSA	1.73 × 10^5^		Static	IIA	2.04	a, c	[171]
Flavonoids	Delphinidin-3-*O*-glucoside	BSA	1.98 × 10^5^	1.100	Static	IIA	2.81	c, d	[116]
		HSA	1.443 × 10^5^	1.082		IIA	2.34	a	[135]
	Pelargonidin-3-*O*-glucoside	HSA	6.89 × 10^4^	0.995	Static, dynamic	IIA	2.83	c, d	[135]
		BSA	1.21 × 10^5^	1.057	Static	IIA	2.74	b	[172]
	Cyanidin-3-*O*-glucoside	HSA	1.096 × 10^5^	1.042	Static, dynamic	IIA	2.58	b	[135]
	Neohesperidin dihydrochalcone	HSA	2.79 × 10^4^	1.02	Static	IIA		c, d	[117]
	Icariin	HSA	3.0335 × 10^4^	1.0282	Static	IIA	2.5	c, d	[133]
	Guaijaverin	HSA	2.02 × 10^5^	1.121	Static	IIA	2.527	a, b, c	[173]
	Mangiferin	BSA	3.18 × 10^4^	1.16	Static	IIA		a, c	[154]
	Berberine	BSA	7.75 × 10^4^	0.92	Static	IIA		b, c, d	[174]
	Palmatine	BSA	7.11 × 10^4^	1.17	Static	IIA		b, c, d	[174]
	Paclitaxel	HSA	3.241 × 10^2^	0.93	Static	IIA	2.23	a	[119]
Alkaloids	Tetrandrine	BSA	1.06 × 10^6^	1.17	Static, dynamic	IIA	1.455	a, b	[120]
		HSA	1.59 × 10^6^	1.21	Static, dynamic	IIA	1.451	a	[120]
	Brucine	BSA	3.47 × 10^5^	1.04	Static	IIA	5.08	a, b	[175]
Triterpenes	Glycyrrhetinic acid	HSA	K1 = 1.65 × 10^5^ K2 = 0.458 × 10^5^	N1 = 0.43 N2 = 0.73	Static	IIA		1. c 2. a	[121]
	Trans-feruloyl maslinic acid	HSA	1.42 × 10^8^	0.6		IB			[129]
Phenyl-propanolds	Ferulic acid	HSA	7.87 × 10^3^		Static	IIIA		c	[118]
	Caffeic acid	BSA	4.264 × 10^5^	1.173	Static		1.86	c	[118]
	Chlorogenic acid	HSA	3.004 × 10^4^	1.14	Static	Site I	3.10	b	[176]
	Paeonol	HSA	4.84 × 10^3^			IIIA		c, d	[85]
	Curcumin	HSA	1.80 × 10^5^	1.14	Static	IIA		c, d	[144]
Other phenolic substances	Salicylic acid	BSA	5.60 × 10^3^	1.85				c, d	[79]
	N-trans-p-coumaroyltyramine	HSA	4.5 × 10^5^	1.538		IIA		a	[128]
	Syringin	HSA	2.97 × 10^4^	1.336	Static, dynamic	IIA	3.15	a	[121]

*K* is equilibrium binding constant. Stoichiometry (N) is the number of binding sites considered for the fit. *r* is the distance between the Trp residue of HSA and the acceptor molecule. Driving force is noncovalent interactions between drugs and biological macromolecules, and a is hydrophobic interaction, b is electrostatic interaction, c is hydrogen bonds and d is van der Walls force

### Binding rules between different types of active herbal components and plasma proteins

Based on the data in Table 2, the properties of different types of active herbal components are significantly different. For the flavonoids, hydrophobic interaction and hydrogen bonds are the major noncovalent interactions between drugs and proteins. Most flavonoid compounds often contain one or more hydroxyl groups, such as C-5 and C-7 in A ring and C-3′, C-4′ and C-5′ in B ring. These hydroxyl groups could form hydrogen bonds with amino acid residue in *α*-helical domains of serum albumin. The major presence of hydrogen bonds in phenylpropanolds may be due to the same reason. Because of the complex structure of alkaloids, the binding forces of them include all the four types. The quenching mechanism of most active herbal components is static. It indicated that complexes can be formed between the active herbal components and plasma proteins, which are conducive to the distribution and pharmacological actions of the active herbal components. The binding site of most active herbal components was site 1 in subdomain IIA of HSA. So the displacement binding of the active herbal components should be taken into consideration in the compatibility of TCMs, so as not to affect the efficacy.

### Influence factors on the interaction between active herbal components and plasma proteins

In practice, because of the complexity and diversity of active components in herbal medicine, an analysis of condition changes of drug binding may promote the understanding of the molecular mechanisms involved and clinical relevance about them. Several factors have significant impacts on the interaction of active herbal components with plasma proteins.

At first, the degree of drug binding to plasma proteins is greatly influenced by pH [33, 177]. Many researches have demonstrated the obvious correlation between the binding degree and pH levels by in vitro assays. By fluorescence quenching method, Cahyana et al. investigated the effects of structure and pH to the constants for binding of anthocyanins and HSA [178]. The range of the binding constants of anthocyanins with HSA was 1.08 × 10^5^ M^−1^ to 13.2 × 10^5^ M^−1^. Due to the special structure of anthocyanin, such as chalcone, hemiacetal, flavyliumcation, quinoidal bases, the binding affinity was pH-dependent. But this dependency was not always positive correlated. Glycosylation and hydroxyl substituents of anthocyanin had lower affinity to HSA at pH 4, but had relatively potent binding at pH 7.4. However, methylation of a hydroxyl group had the opposite conclusion. The phenomenon that many active herbal components are sensitive to pH may be due to the existence of the acidic phenolic hydroxyl groups. The polar and charged amino acid residues on the protein surface could react with the phenolic hydroxyl by a hydrogen interaction. The change of pH could cause different concentrations of the ionization state and then impact the ability of molecules to bind to HSA.

Then, temperature is another main factor in the interaction of active herbal components with plasma protein. The increasing temperature could affect the binding of small molecules and protein for different quenching types. For static quenching, the binding constants decrease with increasing temperature. On the contrary, the binding constants of dynamic quenching process increase with increasing temperature. Cheng et al. demonstrated that the mechanism of the fluorescence quenching of HSA induced by icariin was static quenching [133]. The binding constants were 3.0335 × 10^4^, 2.0165 × 10^4^ and 1.4227 × 10^4^ L mol^−1^ at 298, 304 and 310 K, respectively, in the fluorescence quenching experiments. The trend that the binding constants vary with increasing temperatures is also the judgement standard of the quenching mechanism.

Afterwards, as trace elements in the blood of the human body, metal ions can act on the active center of the enzyme and play an important role in the normal physiological metabolism. The presence of metal ion directly affects the interaction of plasma proteins with active herbal components. Hu et al. investigated the effects of metal ions on chlorogenic acid-HSA system. Since adding the metal ions (La^3+^, Ce^3+^, Fe^3+^, Cr^3+^, Co^2+^, Hg^2+^, Cu^2+^, Mn^2+^, Zn^2+^), the chlorogenic acid-HSA binding constants ranged from 62 to 108% of the value of the CGA-HSA binding constant without ions [176]. Hegde et al. found that the presence of different concentrations of Zn^2+^ decreased the binding constants of hesperitin-HSA [123]. That might be due to the flexible coordination geometry of the metal ion, which allowed the rapid shift conformations of proteins to perform biological reaction.

Also, the binding is associated with the enantioselective interaction between plasma protein and chiral compounds. Two enantiomers of the same chiral compound have different optical properties, physical and chemical properties and biological activities. Stereoselective difference in PPB between clausenamide (CLA) enantiomers has been found by equilibrium dialysis in rat plasma protein binding [179]. The results of this trial indicated that mean percentages of (−) and (+) CLA in the binding form were 28.5 and 38.0%, respectively. The results explained the stereoselective differences in pharmacokinetics in rats by intravenous drip and oral administration trials between CLA enantiomers. Sun et al. investigated the binding of tetrahydropalmatine (THP) enantiomers and HSA, AGP and proteins in human plasma and found that (+)-THP had higher affinity to HSA and AGP than (−)-THP, respectively [180].

Finally, species differences are also a vital factor that can affect the binding of active components binding and plasma proteins. Liu et al. investigated the plasma protein binding rates of naringin and aglycone naringenin in rat, dog and human plasma by equilibrium dialysis combined with LC–ESI–MS/MS. The plasma protein binding ratios of naringin were found to be 83.30–84.56%, 48.17–51.33% and 72.14–74.06% in rat, dog and human plasma, respectively. Gu et al. used equilibrium dialysis followed by LC–MS analysis to assess 20(R)-ginsenoside Rh_2_ plasma protein binding at four concentration levels (50, 100, 200 and 400 ng mL^−1^) in rat and human plasma [181]. They suggested that the binding degrees were about 27% for human plasma and 70% for rat plasma. This diversity indicated that species difference was an inevitable factor in new drug development containing 20(*R*)-ginsenoside Rh_2_. There are differences between different species about plasma protein binding, but they often have a good correlation [182]. Therefore, measuring the drug binding in other species contributes immeasurably to the forecast of human plasma.

### Method selection for different types of active herbal components

The active herbal components in TCMs are complex and the properties of different types of medicines are quite different. Therefore, the selection of appropriate methods for the drug-protein binding studies would be of great significance. Each method has its own advantages and restrictions, and it depends on the situation.

Firstly, researchers should take into consideration the aim and experimental condition. If you just want to get the binding affinity of the active herbal components and plasma proteins, membrane technology, centrifugation, extraction methods and chromatographic methods are the good choice. While in the advanced drug discovery or development stages, chromatographic methods, electrochemical methods and calorimetry are the methods of choice to obtain a complete view of the binding mechanisms [22]. In these methods, equilibrium dialysis and ultrafiltration are the classical detection methods. These methods are cheap, simple to operate and easily available, and thus they are widely used to evaluate the binding of drug and protein in the early phases of drug development.

Another important consideration is the properties of active herbal components. The classical methods (ED, UF, and UC) are suited to investigate most of the water-soluble compounds. If the water solubility of some active herbal components is low, chromatographic methods, electrochemical methods and calorimetry may fail because the compounds need dissolve into phosphate buffered saline at pH 7.4 in these methods. The advantage of HF-LLPME is that the method can be applied to different physicochemical property drugs with different extraction modes, and the sensitivity and reproducibility are comparable. HF-LLPME is suitable for the extraction of samples with high solubility in the organic phase. For the volatile components in Chinese herbal medicine, like volatile oils which give certain herbs their distinctive aroma, conventional techniques could easily lead to the loss of the samples and affect the final results. Headspace-SPME is much suitable for those components.

Then, researchers need to consider the purity and quantity of the active herbal components. For the low purity compounds, CE and HPAC are better choices. Some active herbal components are expensive and scarce. CE inherits its advantages including speediness, low consumption of sample and reagent, high separation efficiency, and availability in the same or similar physiological system conditions. And this method is a good choice for those compounds.

Finally, because of the complexity of the chemical constituents of TCMs, it is important to choose the suitable technique to determine the binding of multi-components in TCMs and plasma proteins simultaneously. HPAC has obvious advantages in investigation of multi-components in TCMs which could react with plasma proteins. Conventional screening methods are blind and massive. HSA or AAG is prepared as the stationary phase of HPAC and affinity chromatography screening model can be established. These components in herbal extracts, which can specifically bind to the receptor protein, are retained in the column, so that the active components in TCMs can be found quickly and effectively. Microdialysis technology can directly obtain the free drug molecules without protein and analyze the concentration of the free drug without pretreatment. Therefore, this method has unique advantages to study the synergistic effect of multi-components, and is a good guidance for the screening of active components in vitro in Chinese herbal medicine. The combination of CE and microdialysis techniques, which inherits the advantages of both methods, could objectively analyze the drug-protein binding behavior of specific drugs under physiological and/or pathological conditions.

## Conclusions

The safety and efficacy of Chinese herbal medicines have been proven through experience passed on from generation to generation in China. Chinese herbal medicines experienced the change from the single herb to the compound medicines under the guidance of TCM theory and had established itself as a relatively independent disciplinary system. The single active component in Chinese herbal medicines has developed into a multitude of new drugs, and artemisinin is a typical example. Therefore, studies on the binding of active components in Chinese herbal medicines and plasma proteins are of great significance to the guidance and evaluation of new drug development. This article reviewed common techniques including membrane technology, centrifugation, extraction methods, chromatographic methods, spectroscopic methods, electrochemical methods and calorimetry. Rules and influence factors of the binding between different types of active herbal components and plasma proteins are summarized in the end. And some suggestions are also given to help to choose the suitable technique.

But holism is a key element of all systems of traditional medicine and compound prescription is the advantage of traditional Chinese medicine. Under compatibility theory of Chinese medicine, the drugs could enhance effect, reduce toxicity, expand treatment coverage and be an effective preparation for the treatment of complex diseases. The complexity of the compositions of the traditional Chinese medicine prescription has become the biggest obstacle to the further development of traditional medicine. And, based on the existing research results, future studies are worthy to be performed to further study the plasma proteins binding rules of the major active components in single herb or Chinese medicine prescription under the guidance of holism and system biology. More new technologies should be used, and the combination of multiple analytical methods is a new trend to study the interaction between active ingredients of traditional Chinese medicine and plasma proteins. And these studies will, hopefully, be guiding factors in futuristic endeavor to scientifically explain the efficacy and the overall mechanism of action of traditional Chinese medicine.

## Abbreviations

TCM: traditional Chinese medicine
PPB: plasma protein binding
ADMET: absorption, distribution, metabolism, excretion and toxicity
HSA: human serum albumin
AAG: *α*_1_-acid glycoprotein
BSA: bovine serum albumin
ED: equilibrium dialysis
HPLC: high performance liquid chromatography
RED: rapid equilibrium dialysis
PAMPA: parallel artificial membrane permeability assay
GC–MS: gas chromatography–mass spectrometry
LC-IT-TOF-MS: liquid chromatography-ion trap-time of flight mass spectrometry
RRLC-ESI–MS–MS: rapid resolution liquid chromatography–electrospray ionization–mass spectrometry
UF: ultrafiltration
CE: capillary electrophoresis
NMR: nuclear magnetic resonance
UC: ultracentrifugation
SPME: solid phase microextration
HF-LLPME: hollow fiber liquid–liquid phase microextraction
LLE: liquid–liquid extraction
HPAC: high performance affinity chromatography
ACE: affinity capillary electrophoresis
VP: vacancy peak
HD: Hummel–Dreyer
FA: frontal analysis
CZE: zone migration capillary electrophoresis
SDS-PAGE: sodium dodecyl sulfate polyacrylamide gel electrophoresis
*Trp*: tryptophan
*Tyr*: tyrosine
*Phe*: phenylalanine
FT-IR: fourier transform infrared spectroscopy
CD: circular dichrosim
SPR: surface plasmon resonance
RLS: resonance light scattering
FRET: fluorescence resonance energy transfer
CV: cyclic voltammetry
ITC: isothermal titration calorimetry
DSC: differential scanning calorimetry
